# Looking back: BMI as a measure of body fatness: age- and sex-specific prediction formulas. Thirty years later

**DOI:** 10.1017/S0007114522000150

**Published:** 2022-04-28

**Authors:** Jacob C. Seidell

**Affiliations:** Department of Health Sciences, Vrije Universiteit Amsterdam, Van der Boechorststraat 7, 1081 BT Amsterdam (room MF-J381), the Netherlands

This paper was based on measurements conducted at the department of human nutrition at the Agricultural University in Wageningen the Netherlands. The original ideas were from Paul Deurenberg who was an assistant professor specialised in methodologies to assess body composition and energy metabolism. Paul Deurenberg was the supervisor of two PhD students: Jan A Weststrate (1985–1988) and me (1983–1986) together with the help of many other colleagues and students performed large series of anthropometric measurements of people from all ages who were enrolled in different projects on body composition. In all, it took almost more than five years to complete all measurements in 1229 individuals. In all of these people we measured, apart from a detailed set of anthropometric measurements, body density by underwater weighing.

At that time, we realized that the BMI (or Quetelet’s Index) had severe limitations when it came to assessing an individual’s body fatness. Not only was the correlation between BMI fatness not the same across different age groups but also due to differences in body composition between males and females, and the age-related increase in body fat mass and the decrease in fat-free mass the relationship between BF% and BMI should be sex- and age-dependent.

There were, however, at that time no equations from which one could assess body fat percentage from BMI.

Inspired by the paper by Durnin and Womersley^(1)^, who published equations from which body fatness (as measured by body densitormetry) could be estimated from skinfolds we decided to develop such equations for the BMI.

## What did the paper tell us and why is it so highly cited?

In children aged between 4 and15 years, the body fat percentage (BF%) could be predicted by the formula BF% = 1·51 × BMI–0·70 × age–3·6 × sex + 1·4. Sex was scored as zero for women and one for men. This equation implies that at a given BMI and age, boys have a 3·6 percent point lower body fat percentage than girls and that for a given BMI and sex older children have a lower fat percentage than younger children. The explained proportion of variance of body fat percentage by this equation was, however, relatively low (38 %).

In adults, the prediction formula was BF% = 1·20 × BMI + 0·23 × age–10·8 × sex–5·4. Again, sex was scored as zero for women and one for men.

This implies that for a given BMI and age, men have a 10·8 percent point lower body fat percentage compared with women and that body fatness at a given BMI increases with age. In contrast to children, this equation explained a large proportion of the variance (79 %).

The correlations between BMI and body fat percentage differed greatly by sex and age. The lowest correlation was in adolescent boys aged 16–20 years (*r* = 0·39) and men aged 66 years and older (*r* 0·37. The highest correlations were observed in men and women aged 26–35 years (*r*−0·92 and 0·89, respectively).

The equations were cross-validated (they were constructed from data in half the sample and validated in the other half).

A somewhat unexpected finding was not only an inverse relation between BMI and height in adults but also between body fat percentage and height. This may well have been an effect of age because older adults tended to be shorter and had a relatively high BMI and body fat percentage. But it does suggest that the presumption that an index based on height and weight needs to be independent of height may not be true.

The paper continues to be highly cited. On January 5th, it was cited 806 times according to the Web of Science and 1840 times according to Google Scholar. Remarkably, the yearly number of citations increased over time and reached its peak in 2017 (132 citations in Google Scholar in that year; 26 years after its publication). The reason the paper is highly cited may be the widespread use of the BMI and the need to translate BMI values into levels of fatness. The paper is also often cited in the context of the low correlation between BMI and body fatness in specific age groups (particularly in growing children and older people).

## What happened next?

Paul Deurenberg continued to perform body composition research. First in Wageningen and subsequently in Singapore where he particularly focused on the ethnic differences in body composition and health of the local population (Malay, Chinese and Indian). He suggested that ethnic-specific criteria are needed to specify levels of fatness and associated cardiometabolic health indicators^(2)^.

Jan A. Weststrate pursued a career in research and development in the food industry (in companies such as Unilever, PepsiCo). From 1983 to about 2002, I focused much on the importance of body fat distribution to health rather than overall body fat. Particularly I studied the relation between abdominal (visceral) fat and health before I moved into public health research. I coauthored two recent position statements that focused on the importance of looking at specific fat depots in the body rather than total body fatness^(3,4)^. But despite the general recognition that body fat distribution is critically important in cardiometabolic health, the BMI continues to be used widely in clinical practice and epidemiological research to evaluate adipose tissue accumulation-related health risks.

## References

[1] Durnin JVGA & Womersley J (1974) Body fat assessed from total body density and its estimation from skinfold thickness: measurements on 481 men and women aged from 16 to 72 Years. Br J Nutr 32, 77–97. 4843734 10.1079/bjn19740060

[2] Deurenberg-Yap M , Schmidt G , van Staveren WA , et al. (2001) Body fat measurement among Singaporean Chinese, Malays and Indians: a comparative study using a four-compartment model and different two-compartment models. Br J Nutr 85, 491–498. 11348564 10.1079/bjn2000276

[3] Ross R , Neeland IJ , Yamashita S , et al. (2020) Waist circumference as a vital sign in clinical practice: a Consensus Statement from the IAS and ICCR Working Group on Visceral Obesity. Nat Rev Endocrinol 16, 177–189. 32020062 10.1038/s41574-019-0310-7 PMC7027970

[4] Neeland IJ , Ross R , Després JP , et al. (2019) Visceral and ectopic fat, atherosclerosis, and cardiometabolic disease: a position statement. International Atherosclerosis Society; International Chair on Cardiometabolic Risk Working Group on Visceral Obesity. Lancet Diabetes Endocrinol 7, 715–725. 31301983 10.1016/S2213-8587(19)30084-1

